# Nursing surgery in late preterm

**DOI:** 10.1186/1824-7288-40-S2-A45

**Published:** 2014-10-09

**Authors:** Chiara Selmi

**Affiliations:** 1 U.O. PEDIATRIA , USL 4 PRATO, 59100 Italy

## 

Nursing surgery born for an organizational and strategical choise which allowed us to create a link whit hospital and territorial children’s doctor, going to fill the lack of assistance during the period of hospital discharge and first visit of specialist for children’s. Our aims are essentially two: see to peaceful back home baby and their family, insure a continuity of care ,continuing the technical educational intervention started during hospitalization, increasing the confidence in themselves in this new role. This anamnestic assessment carried out together with the parents enables us to empathize with the parent to allow itself to expose their doubts and questions. The cases to which addressed a nurse’s control are: weight loss >10%, maternal-fetal incompatibility group, deficit of G6PDH (Glucose-6-phosphate dehydrogenase), values predischarge of bilirubins in the intermediate zone of risk such as descript of AAP guidelines, baby with perinatal risk factors of infection, preterm infants, SGA (Small for Gestational Age) infants, infants from families at risk (drug addiction, alcoholism, poor socio-economic conditions, migrant families without residence permits) , red highlights not executed due to technical problems at birth (eyelids tightened); doubt audiological screening at birth, any other baby who, for whatever reason, the physician who performs the discharge decides to revalue in post discharge. Access to the surgery is by reservation CUP that takes place directly at the discharge of the baby and is closely related to the hospital stay and do not in any way replace the first visit to the pediatrician to be made within 7° to 10° days after discharge.

